# Neuroimaging of acute myocardial injury in stroke: insights into brain lesion locations and network disconnections

**DOI:** 10.3389/fneur.2026.1719600

**Published:** 2026-02-11

**Authors:** Petr Mikulenka, James W. Garrard, Olivia N. Murray, Alistair Perry, George Harston, Michal Mihalovič, Petr Toušek, Tomáš Peisker, Ivana Štětkářová, Davide Carone

**Affiliations:** 1 Department of Neurology, Third Faculty of Medicine, Charles University and University Hospital Královské Vinohrady, Prague, Czechia; 2 Brainomix Limited, Oxford, United Kingdom; 3 Radcliffe Department of Medicine, University of Oxford, Oxford, United Kingdom; 4 Oxford University Hospitals NHS Foundation Trust, Oxford, United Kingdom; 5 Division of Informatics, Imaging and Data Sciences, University of Manchester, Manchester, United Kingdom; 6 Department of Cardiology, Third Faculty of Medicine, Charles University and University Hospital Královské Vinohrady, Prague, Czechia

**Keywords:** autonomic nervous system, brain connectivity, myocardial injury, neurocardiology, stroke, lesion-symptom mapping, troponin

## Abstract

**Introduction:**

Acute myocardial injury (AMI) is frequently observed in patients with acute stroke and may be linked to lesion location within the central autonomic network (CAN). However, neuroimaging findings remain inconsistent, and the neuroanatomical basis remains unclear. We investigated associations between lesion location, structural disconnection, and AMI in acute stroke.

**Methods:**

In this single-center cohort, patients with ischemic or hemorrhagic stroke were screened for AMI using serial high-sensitivity cardiac troponin I (hs-cTnI) measurements within 48 h of admission. AMI was defined as hs-cTnI levels exceeding the 99th percentile of the reference limit with >20% dynamic change. Stroke lesions were segmented using Brainomix software and overlaid on a diffusion tensor imaging template to generate indirect structural disconnection maps. A voxel-based multivariate method (Sparse Canonical Correlation Analysis) identified associations between AMI and (a) directly damaged areas and (b) disconnected regions.

**Results:**

Among 281 patients (mean age 71.4 ± 12.3 years), 59 (21%) developed AMI. AMI was associated with lesions in the left insula, basal ganglia, and adjacent white matter, as well as with widespread structural disconnection involving CAN regions. It was also significantly associated with older age, female sex, and higher NIHSS scores, but not with atrial fibrillation, diabetes, or hypertension.

**Conclusion:**

In this cohort, AMI affected 21% of stroke patients and was significantly associated with specific lesion locations and widespread structural disconnection, including regions implicated in autonomic regulation, but not with traditional cardiovascular risk factors. These findings support the hypothesis that stroke-induced autonomic dysregulation may contribute to AMI development.

## 1 Introduction

Adverse cardiac events frequently occur in patients suffering from ischemic or hemorrhagic stroke (1–4). For early detection of these complications, guidelines recommend baseline troponin evaluation in both types of stroke (5, 6). While elevated baseline troponin levels have been extensively studied and consistently linked to a worse prognosis, including poorer functional outcomes and increased mortality (7, 8), dynamic changes indicating concomitant acute myocardial injury have been less studied in acute stroke patient cohorts (9–11). Therefore, the characteristics and underlying pathophysiology of acute myocardial injury remains unclear.

Several mechanisms have been proposed to underlie acute myocardial injury after acute stroke, including atherothrombotic myocardial infarction (type 1 myocardial infarction), demand ischemia (type 2 myocardial infarction), microcirculatory dysfunction (cardiac syndrome X), nonischemic causes such as myocarditis or Takotsubo syndrome, and systemic conditions such as sepsis or pulmonary embolism (12). Recently, the concept of Stroke-Heart Syndrome has emerged, suggesting that stroke-induced stress exacerbates cardiac injury through autonomic dysregulation with local and systemic inflammation, potentially leading to a broad spectrum of cardiac complications, including arrhythmias and both ischemic (e.g., type 1 and type 2 myocardial infarction and neurocardiogenic myocardial necrosis) and nonischemic acute myocardial injuries (e.g., Takotsubo syndrome) (4, 13–17).

Available evidence from clinical and preclinical research suggests that specific stroke lesion locations may increase the risk of myocardial injury, potentially by triggering autonomic dysregulation (13, 18). While previous studies have particularly emphasized lesions in the right insula, a key component of the central autonomic network (CAN), lesions in the left insula and right inferior parietal lobe have also been implicated in myocardial injury (19–22). Conversely, other studies have reported no significant association between stroke lesion location and myocardial injury (23–25). We hypothesize that acute myocardial injury may result not only from localized brain damage but also from structural disconnections within the CAN. However, the relationship between acute myocardial injury and stroke lesions has not yet been examined using a structural disconnectome approach. Consequently, the neuroanatomical basis of stroke-related myocardial injury remains incompletely understood. Exploring structural network disconnections may reveal insights beyond those provided by traditional lesion-symptom mapping.

In this study, we aimed to investigate the associations between lesion location, structural disconnection, and acute myocardial injury in acute stroke patients. We also assessed the prevalence and risk factors for acute myocardial injury, including demographic characteristics, cardiovascular history, stroke severity, and acute revascularization treatments.

## 2 Methods

We prospectively recruited consecutive patients with acute ischemic or hemorrhagic stroke admitted to the stroke unit of a tertiary care hospital over a 35-month period (August 2020–July 2023). The study was approved by the Ethics Committee of our institution (University Hospital Královské Vinohrady, Prague, Czech Republic, No. EK-VP/531/1/2020), and informed consent was obtained from all participants.

**Figure 1:**
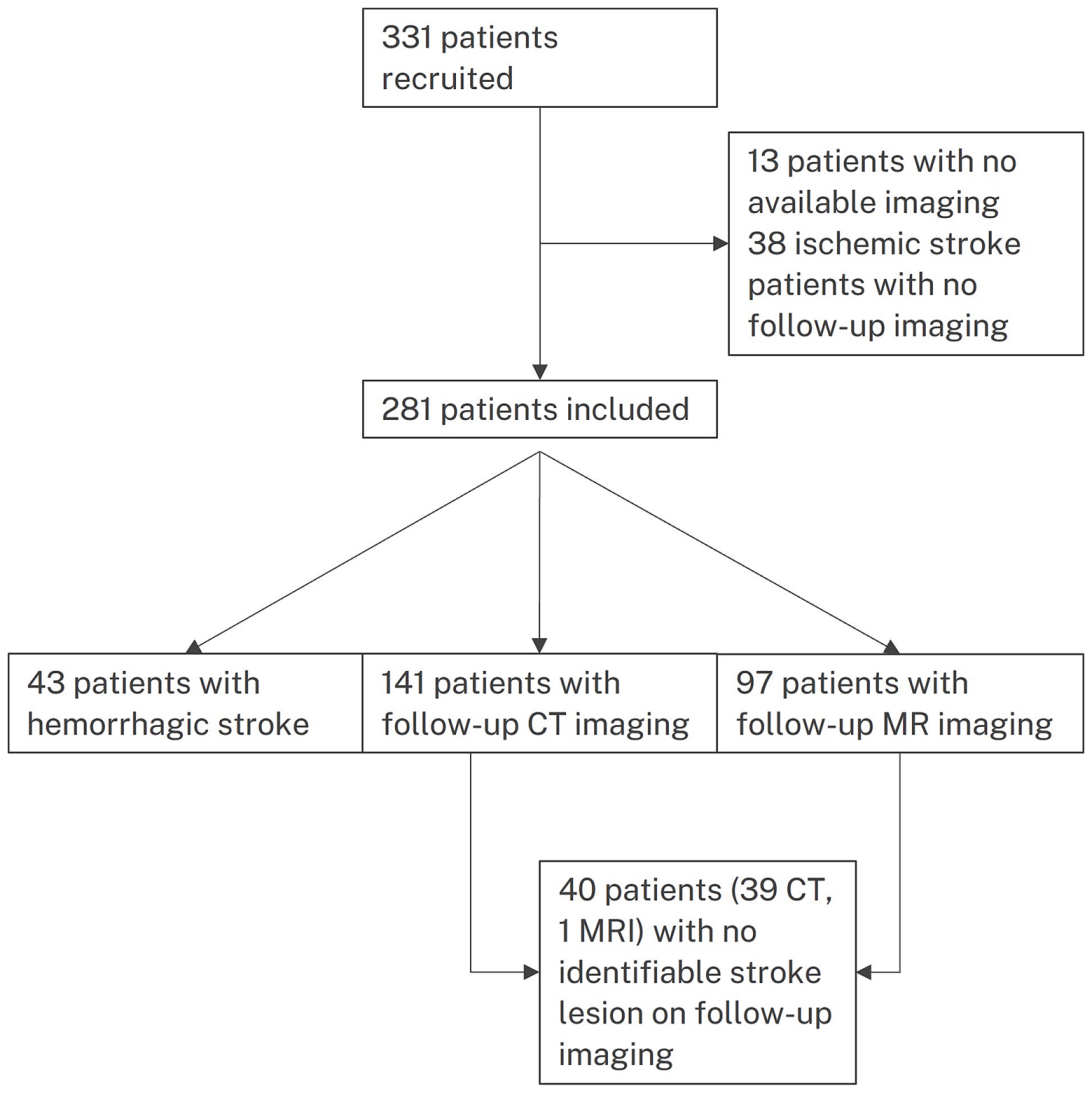
Flowchart of patients.

The diagnosis of stroke was based on clinical presentation and radiological evidence of ischemic or hemorrhagic stroke, with acute lesions identified on non-contrast head CT (NCCT), perfusion CT, or MRI. Patients with ischemic stroke underwent follow-up imaging using CT or MRI. The flowchart of patient inclusion is presented in Figure 1.

Enrolled patients were screened for acute myocardial injury using serial high-sensitivity cardiac troponin I (hs-cTnI) measurements, taken at admission and repeated during routine morning blood sampling (typically around 06:00) on the following 2 days to ensure serial monitoring within the first 48 h. In accordance with the Fourth Universal Definition of Myocardial Infarction (26) and as described previously (10), myocardial injury was classified as acute if hs-cTnI levels exceeded the 99th percentile of the upper reference limit (URL) (cut-offs at our institution: 53 ng/L for men and 34 ng/L for women) along with a > 20% rise or fall in subsequent troponin concentrations.

Patient demographics, medical history, clinical data including stroke characteristics (NIHSS, mRS), stroke treatment details (intravenous thrombolysis or endovascular therapy) were collected.

### 2.1 Imaging analysis

All brain imaging was analyzed with Brainomix in-house software. For patients with ischemic stroke, the follow-up infarct region of interest was automatically segmented using published Brainomix software (26). The resulting segmentation was then manually reviewed and edited where necessary by an experienced stroke fellow. MRI follow-up imaging using an ischemic stroke lesion segmentation model based on diffusion-weighted imaging (*b* = 1,000 s/mm^2^) and apparent diffusion coefficient was used when available. If only CT follow-up brain imaging was available, then this was used. For patients with intracerebral hemorrhage, lesion segmentation was based on the baseline NCCT scan. These were processed automatically using Brainomix 360 Stroke software, an FDA-cleared and CE-marked decision support tool that assesses stroke signs on CT scans to generate a hemorrhage volume mask for each case, limited to areas of intraparenchymal hemorrhage only (27). While hematoma expansion can occur during the early phase, baseline imaging is routinely used in stroke studies to estimate initial hematoma volume and location, and follow-up imaging was not systematically available in our cohort. The stroke lesions were registered into MNI standard space, using non-linear registration (FSL), a standard approach to perform group-level analysis (28).

#### 2.1.1 Disconnection maps

Disconnection maps were generated using the Brain Connectivity and Behavior Toolkit (BCBToolkit). This method combines DTI data from 47 healthy controls—who were not matched to our study population—with patients’ lesion maps as seed regions for tractography to estimate structural disconnection, even without study-specific DTI data. It calculates the probability of each voxel being disconnected due to the stroke lesion, producing a spatial map for each patient that indicates disconnection likelihood on a scale from 0 to 1. The maps were thresholded at the BCBToolkit default value of 0.5 and subsequently binarized (29). Given the paucity of MRI and absence of diffusion tensor imaging, it was not feasible to rely on a study-specific template, and hence this external template was used.

#### 2.1.2 Lesion and disconnection symptom mapping

The relationship between both the co-registered stroke lesions and disconnection maps to the clinical outcomes was analyzed using the sparse canonical correlation analysis (SCCAN) technique. This method is a modified form of principal component analysis which is multivariate, with improved accuracy over conventional univariate models which adjusts for lesion size, and is implemented using the LESYMAP package^1^ in the R-toolbox (30). The output of this technique is a population-level estimation of the likelihood of each voxel being associated with each of the clinical outcomes of interest.

A separate LESYMAP analysis was performed on both the lesions and disconnection maps to generate two spatial maps for the primary outcome of acute myocardial injury. A 10% threshold for each voxel involvement was used for each analysis, the default setting in LESYMAP.

In addition, to explore whether the disconnectome patterns associated with stroke severity differed from those linked to acute myocardial injury, an identical analysis was also performed using NIHSS scores as a secondary outcome, generating lesion and disconnection maps for comparison.

#### 2.1.3 Atlas overlap

The volume of overlap between the output from the lesion symptom mapping software with two atlases, JHU (31) and AAL3 (32), was calculated. This was performed for both the areas of lesion and disconnection associated with acute myocardial injury.

#### 2.1.4 Subgroup analyses

Subgroup analysis of ischemic stroke patients and hemorrhagic stroke patients was conducted to evaluate the association between acute myocardial injury for both lesion involvement and disconnection.

Given the likely underlying heterogeneity in the locations of the stroke lesions in this cohort, it was planned that the lesion-symptom mapping would be repeated at a lower 5% threshold, if no significance was found at a 10% threshold.

### 2.2 Statistical analysis

The Mann–Whitney *U* test was employed for comparing median values across groups, while the Chi-squared test was used to evaluate associations within categorical data. As the comparisons in Table 1 were exploratory in nature, no correction for multiple comparisons was applied. Therefore, *p*-values are reported for descriptive purposes only.

**Table 1:** Differences in characteristics in patients with acute myocardial injury.

Variable	Description	Acute myocardial injury (*n* = 59)	No acute myocardial injury (*n* = 222)	*p* -value
Age	Median (IQR)	77 (14)	70 (16.75)	<0.001
Female Sex	% (*n*)	63% (37/59)	42% (92/221)	0.004
Admission NIHSS	Median (IQR)	11 (11)	8 (10)	0.009
Hypertension	% (*n*)	76% (45/59)	76% (166/219)	0.94
Diabetes	% (*n*)	32% (19/59)	22% (47/218)	0.089
Ischemic heart disease	% (*n*)	17% (10/58)	6% (14/219)	0.009
Previous stroke	% (*n*)	5% (3/59)	7% (16/219)	0.548
Smoking history	% (*n*)	38% (22/58)	43% (92/213)	0.472
LV impairment	% (*n*)	8% (4/48)	4% (7/190)	0.17
Previous MI	% (*n*)	11% (6/57)	3% (7/219)	0.02
Known AF	% (*n*)	25% (15/59)	20% (45/222)	0.391
AF detected after stroke	% (*n*)	15% (9/59)	18% (39/222)	0.675
Hemorrhagic stroke	% (*n*)	14% (8/59)	15% (34/221)	0.727
Thrombolysis	% (*n*)	71% (42/59)	60% (132/221)	0.107
Thrombectomy	% (*n*)	40% (23/58)	25% (54/216)	0.027
Size of stroke	Median (IQR)	26.8 (61.7)	3.4 (23.1)	0.017
Stroke location	Left	27 (45.7%)	86 (38.7%)	0.616
	Right	25 (42.4%)	105 (47.3%)	
	Posterior	7 (11.9%)	31(14.0%)	

## 3 Results

### 3.1 Patient characteristics and prevalence of acute myocardial injury

The study cohort included 281 stroke patients (median age 72 years, 46% were female), comprising 238 ischemic and 43 hemorrhagic strokes. Hypertension was present in 76%, atrial fibrillation in 39%, and ischemic heart disease in 9%. Thrombolysis was performed in 62%, and thrombectomy in 28%. A full summary of patient demographics is provided in Supplementary Table 1.

Acute myocardial injury occurred in 59 (21%) of patients. Table 1 shows the differences in patient characteristics between those with and without acute myocardial injury. This included older age, higher proportion of female sex, higher stroke severity (NIHSS score), ischemic heart disease, previous myocardial infarction, and higher rates of thrombectomy, and larger stroke volumes compared to patients without acute myocardial injury. There was no difference in the stroke hemispheres between groups.

Among patients with acute myocardial injury, 8/59 (13.6%) were diagnosed with acute myocardial infarction by the treating cardiology team (1x ST-segment elevation myocardial infarction, 7 x non–ST-segment elevation myocardial infarction). Coronary angiography was performed in 3/59 (5.1%) cases, demonstrating single-vessel disease in 2 patients and three-vessel disease in 1 patient.

### 3.2 Lesion mapping: brain regions associated with acute myocardial injury

**Figure 2:**
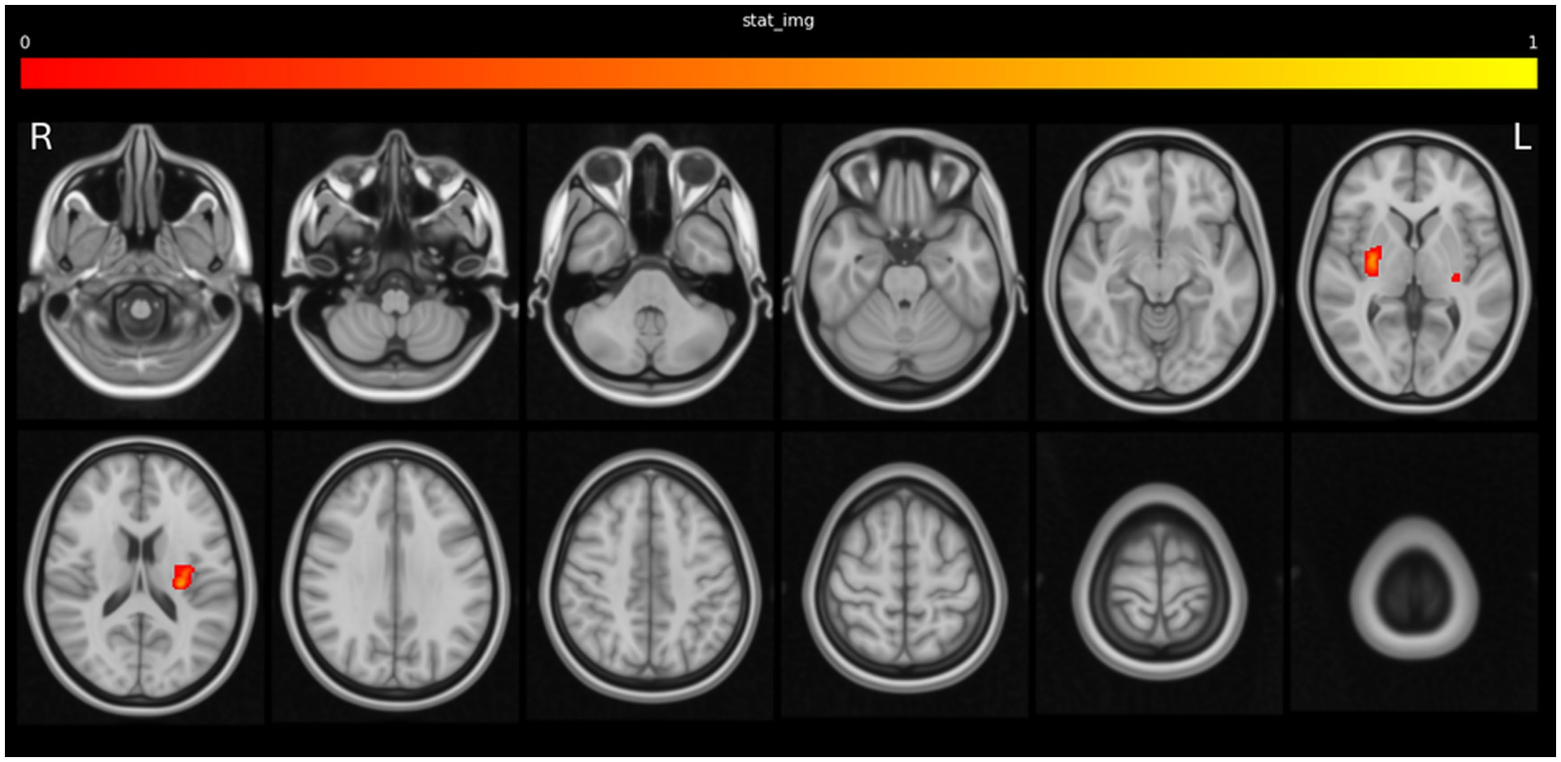
Lesion locations associated with acute myocardial injury.

The frequency map of stroke lesion locations across the whole population is shown in Supplementary Figure 1, with most strokes in the anterior circulation. There was a statistically significant association between stroke lesions in the left insula and bilateral basal ganglia and surrounding white matter with the occurrence of acute myocardial injury. This is demonstrated in Figure 2, Supplementary Table 2 and in 3D in Supplementary Video 1.

When restricting the analysis to include only ischemic stroke patients with evidence of a lesion, the association between involvement of the right basal ganglia and acute myocardial injury remained statistically significant (Supplementary Figure 2A). There were no significant associations seen when restricting the analysis to hemorrhagic stroke patients.

### 3.3 Structural disconnection patterns associated with acute myocardial injury

**Figure 3:**
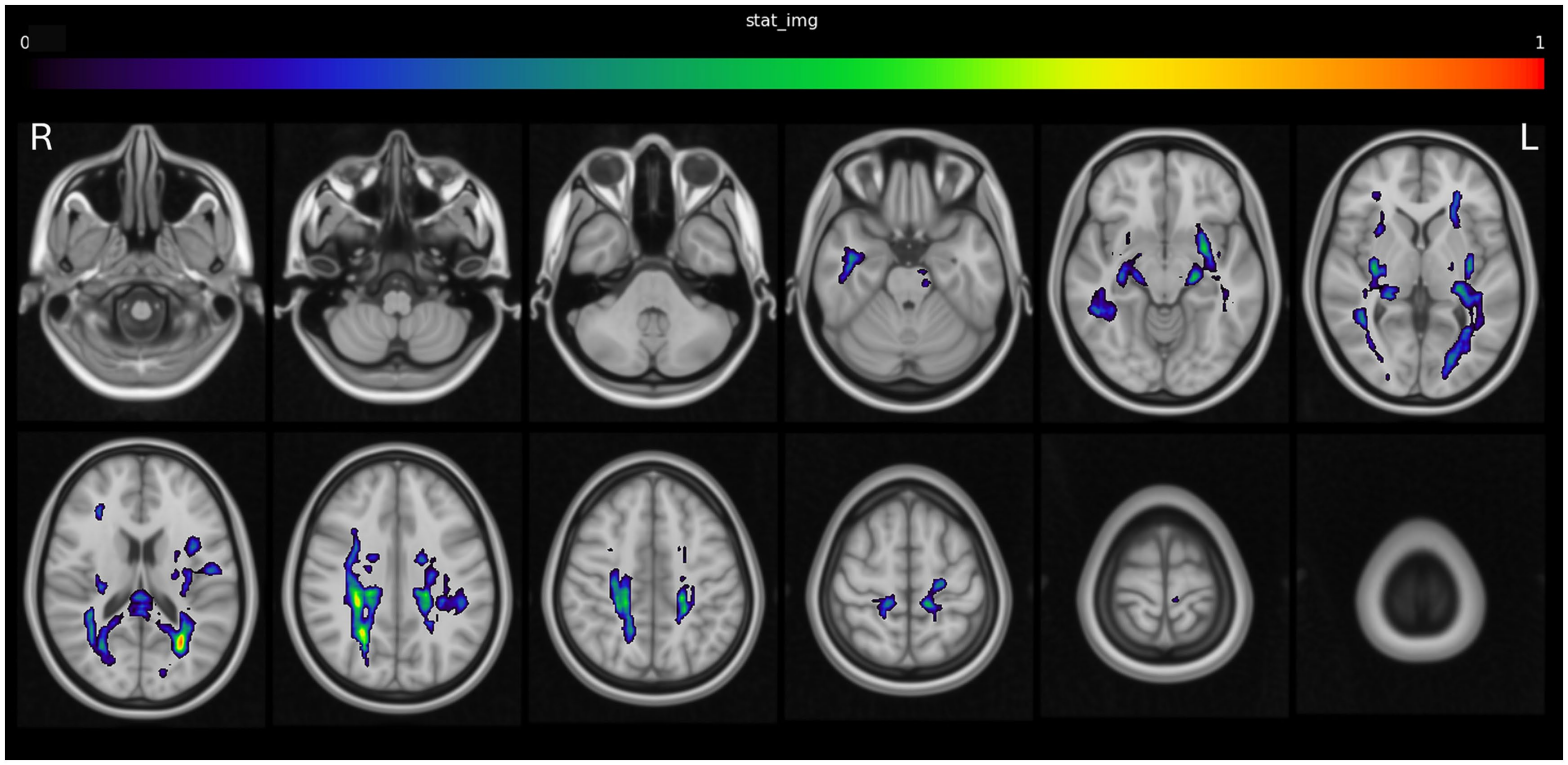
Structural disconnection associated with acute myocardial injury.

A statistically significant relationship was observed between disconnection of regions including the posterior cingulate cortex, hippocampus, corpus callosum, thalamic radiations, superior longitudinal fasciculus, and inferior fronto-occipital fasciculus, and the occurrence of acute myocardial injury. This is demonstrated in Figure 3, Supplementary Table 3 and in 3D in Supplementary Video 2.

A similar pattern, albeit with some predominance to the left brainstem and right cortex, was visible in the subgroup of ischemic stroke patients with a visible lesion on follow-up imaging (Supplementary Figure 2B).

### 3.4 Lesion and disconnection correlates of stroke severity

Supplementary Figures 3A,B demonstrate the relationships of lesion location (3A) and structural disconnection (3B) with stroke severity (NIHSS). Stroke lesions within the basal ganglia bilaterally were associated with increased stroke severity (NIHSS). This spatial pattern was more symmetrical than the relationship between acute myocardial injury and stroke location. Regions of disconnection predominantly within the left hemisphere and corpus callosum were associated with total NIHSS.

## 4 Discussion

Our study identifies four key findings. First, over 21% of acute stroke patients in our cohort (including both ischemic and hemorrhagic stroke) met biomarker-based criteria for acute myocardial injury shortly after admission (≤48 h). Second, the occurrence of acute myocardial injury was significantly associated with older age, female sex, and greater stroke severity, as measured by the NIHSS score and larger strokes. Third, stroke lesions in the left insula and bilateral basal ganglia and surrounding white matter were associated with the occurrence of acute myocardial injury. Fourth, structural disconnection in several brain regions, including the posterior cingulate cortex and precuneus, was linked to acute myocardial injury.

The incidence of acute myocardial injury in acute ischemic stroke patients aligns with findings from previous studies, where the incidence of acute myocardial injury or dynamic troponin changes ranges from 13 to 30.7% (9, 10, 33, 34). However, in hemorrhagic stroke patients, the incidence of acute myocardial injury is slightly lower than that reported by others (11), likely due to the smaller sample size and the inclusion of milder hemorrhagic stroke cases in our cohort. Nevertheless, our findings underscore the high frequency of cardiac complications.

In line with previous findings, acute myocardial injury was linked to higher NIHSS, older age, and female sex (10, 34). While acute myocardial injury was associated with a history of myocardial infarction, it showed no connection to other common cardiovascular risk factors such as hypertension, diabetes, atrial fibrillation, or smoking. These results suggest that the pathophysiology of stroke-related acute myocardial injury might be more complex, potentially involving stroke-induced autonomic dysregulation. The high incidence of acute myocardial injury supports an individualized diagnostic and treatment strategy, as stroke-specific standards for interpreting and managing troponin elevation remain limited. In practice, if the initial hs-cTn is above the upper reference limit, serial troponin measurements should be obtained to confirm AMI and then interpreted in the context of clinical status, ischemic ECG changes, and early echocardiography; in selected patients, this stepwise assessment should prompt advanced cardiac imaging and—when indicated—diagnostic coronary angiography (12). At the same time, in patients with severe neurological deficits (high NIHSS), individualized management should be aligned with the realities of intensive early rehabilitation and may include selected adjunctive modalities such as neuromuscular electrical stimulation (NMES) when appropriate (35).

Using a published voxel-lesion symptom mapping approach (36), in the primary analysis including both ischemic and hemorrhagic strokes, we found that lesions within the left insula, bilateral basal ganglia, and surrounding white matter were significantly associated with acute myocardial injury. While previous neuroimaging studies in ischemic stroke have predominantly highlighted the role of the right insula in patients with elevated troponin levels (19, 22), evidence also suggests that the left insula or bilateral insular cortex lesions contribute to post-stroke cardiovascular complications including baseline troponin elevation (21, 37, 38). Regardless of laterality, the insular cortex is thought to be a critical component of the central autonomic network (39, 40). Additionally, we identified lesions in the right putamen to be associated with acute myocardial injury. Although the putamen is not conventionally classified as a core component of the central autonomic network (CAN), evidence suggests its involvement in autonomic regulation in healthy individuals, as well as its contribution to autonomic dysfunction in conditions such as stroke, Parkinson’s disease, and obstructive sleep apnea (41–45). Furthermore, we demonstrated that lesions in both the internal and external capsules were also associated with acute myocardial injury. While these regions have not been typically linked to autonomic functions, lesions which include a broader set of areas including the insula and the internal and external capsules have been associated with alterations in autonomic modulation in multiple sclerosis (46). We speculate that lesions in these regions may contribute to acute myocardial injury through mechanisms involving autonomic imbalances, consistent with the concept of Stroke-Heart Syndrome (4, 13–15). The spatial pattern of our findings was consistent when restricting the analysis to ischemic stroke patients only. In contrast, no significant voxel-wise associations were detected in the hemorrhagic stroke subgroup. While this may partly reflect limited statistical power, it also aligns with a recent larger ICH cohort in which no specific brain regions were associated with acute myocardial injury, whereas midline shift was identified as the sole independent imaging predictor (47).

In addition to the direct effects of lesions, our findings reveal that structural disconnections extending beyond the lesion site are associated with acute myocardial injury. To our knowledge, this is the first study to employ a structural disconnectome approach in this context, uncovering a widespread pattern of reduced structural connectivity across various gray matter regions and white matter tracts. These disconnections were not restricted to areas belonging to the autonomic system but also affected regions responsible for sensory integration, motor coordination, and visual processing. These findings might explain why patients with acute myocardial injury presented with more severe neurological deficits compared to patients without acute myocardial injury. Importantly, the spatial patterns associated with acute myocardial injury differed from those associated with stroke severity (NIHSS), suggesting that our results are not just a reflection of associations with stroke severity.

While structural connectivity analyses are typically used to assess outcomes in networks with well-defined pathways, such as the corticospinal or visual tracts (48–50), we successfully applied this approach to identify disruptions within the complex and distributed CAN (51–54). Structural disconnections were observed in the posterior cingulate cortex and precuneus, which have also been linked to parasympathetic regulation (51). Additionally, the role of the precuneus in post-stroke cardiac complications has been demonstrated in a study linking lesions in several regions, including the precuneus and hippocampus, to the occurrence of arrhythmias (55). Our findings of decreased connectivity in the hippocampus also align with previous reports of reduced functional connectivity and volume of the hippocampus in patients with Takotsubo syndrome (TTS) (56, 57). We also detected decreased connectivity in the inferior fronto-occipital fasciculus which is identified as part of the occipito-posterior-temporo-frontal pathway within the cortical autonomic network. It is highlighted for its role in connecting the lingual gyrus, insula, and orbitofrontal cortex, which are integral to visceral and emotional autonomic processing (52).

Furthermore, we observed a lateralized pattern in both lesion distribution and structural disconnection, with a predominant impact on the right hemisphere. While both hemispheres were affected, potentially causing autonomic imbalance through disruptions in both the sympathetic and parasympathetic systems, the observed asymmetry aligns with prior evidence suggesting that right hemisphere, particularly right insular, lesions are more strongly associated with pathological sympathetic overactivity (58). This overactivity may trigger a catecholamine surge, leading to myocardial effects such as contraction band necrosis and tachyarrhythmias, resulting in acute myocardial injury (14).

## 5 Limitations

Our study has several limitations. First, the sample size was moderate, and we merged data from both ischemic and hemorrhagic stroke patients, which may introduce pathophysiological heterogeneity. Associations were consistent in ischemic stroke but not significant in the smaller ICH subgroup. Therefore, our lesion-location findings should be interpreted primarily in the context of ischemic stroke. Second, the imaging analysis primarily relied on CT scans as MRI was available in only one-third of cases. While this limits the precision of lesion delineation, it mirrors the clinical reality where MRI is often not feasible in the acute setting. Third, due to the lack of diffusion MRI data, we employed indirect structural disconnectome mapping based on a normative DTI dataset. While this method allows for estimation of structural disconnection, it carries inherent limitations and requires validation against direct tractography methods, and thus warrants careful interpretation (59, 60). Furthermore, serial troponin measurements were obtained at intervals longer than 1–6 h typically recommended for suspected myocardial infarction (61). However, in the context of acute stroke, there are no validated guidelines for troponin timing (14, 62). Prior studies suggest that troponin levels peak approximately 48 h after stroke onset, following a slower trajectory than in acute coronary syndrome (3, 13, 63). Accordingly, this longer sampling window mirrors approaches used in recent large stroke cohorts, where troponin is measured at baseline and reassessed the next day or within 48 h (10, 11). We therefore adopted a similar strategy. Lastly, because cardiac troponin indicates myocardial injury rather than its cause, we could not distinguish stroke-triggered acute myocardial injury from coexisting primary cardiac pathology. In particular, myocardial infarction subtype (e.g., type 1 vs. type 2) could not be systematically classified, as coronary angiography was performed selectively based on clinical indication. Thus, our findings should be considered exploratory and require additional evidence from independent analyses.

## 6 Conclusion

Acute myocardial injury occurred in 21% of patients in our stroke cohort and was associated with specific lesion locations and widespread structural disconnection, including regions implicated in autonomic regulation, with spatial associations most consistent in ischemic stroke. These findings support the hypothesis that stroke-induced disruption of central autonomic pathways, rather than conventional cardiovascular risk factors alone, may contribute to autonomic imbalance and the development of acute myocardial injury. Further research is needed to validate these associations, clarify the underlying mechanisms, and determine whether targeting autonomic dysfunction could help reduce cardiac complications after stroke.

## Data Availability

The raw data supporting the conclusions of this article will be made available by the authors, without undue reservation.

## Footnotes

1
https://github.com/dorianps/LESYMAP
