# Supplementary material for: Neuroimaging of acute myocardial injury in stroke: insights into brain lesion locations and network disconnections
Source: Front Neurol. 2026 Feb 11;17:1719600. doi: 10.3389/fneur.2026.1719600 (PMC12932174; doi:10.3389/fneur.2026.1719600)
Supplement: Supplementary file 1 [file Supplementary_file_1.docx]

Supplementary Material

# **Supplementary Material**

Supplementary video 1: demonstrating the correlation between stroke lesion and acute myocardial injury

Supplementary video 2: demonstrating the correlation between disconnection areas and acute myocardial injury.

Supplementary Figure 1. Spatial location of stroke location across the entire population. Frequency map scale demonstrated from 1 – 25% of patients.

Supplementary Figure 2. A) Images of association between positive infarct voxels and acute myocardial injury. B) Image of the association between the disconnectome associated with positive infarct voxels and acute myocardial injury.

Supplementary Figure 3. Association of lesion voxels (A) and disconnection voxels (B) with NIHSS.

| **Participants** | n |  | 281 |
| --- | --- | --- | --- |
| **Age** | Median (IQR) |  | 72 (62 to 79) |
| **Female Sex** | %(n) |  | 46% (129/281) |
| **Admission NIHSS** | Median (IQR) |  | 8 (5 to 15) |
| **Hypertension** | %(n) |  | 76% (212/279) |
| **Diabetes** | %(n) |  | 24% (66/278) |
| **Ischemic Heart Disease** | %(n) |  | 9% (24/278) |
| **Previous Stroke** | %(n) |  | 7% (20/279) |
| **Smoking** | %(n) |  | 42% (115/272) |
| **LV Impairment** | %(n) |  | 5% (11/239) |
| **History of MI** | %(n) |  | 5% (13/277) |
| **Hemorrhagic Stroke** | %(n) |  | 15% (43/281) |
| **Known AF** | %(n) |  | 22% (61/281) |
| **AF detected after stroke** | %(n) |  | 17% (48/281) |
| **Acute Myocardial injury** | %(n) |  | 21% (59/281) |
| **Thrombolysis** | %(n) |  | 62% (175/281) |

Supplementary Table 1: Demographics of entire patient population

| ROI NAME | ROI VOLUME | LESION OVERLAP VOLUME | LESION % ROI VOLUME |
| --- | --- | --- | --- |
| Putamen_R | 8496 | 288 | 3,389830508 |
| External capsule L | 3600 | 112 | 3,111111111 |
| Retrolenticular part of internal capsule L | 2488 | 64 | 2,572347267 |
| External capsule R | 3728 | 56 | 1,502145923 |
| Posterior limb of internal capsule L | 3816 | 56 | 1,467505241 |
| Inferior fronto-occipital fasciculus R | 2104 | 8 | 0,380228137 |
| Pallidum_R | 2240 | 8 | 0,357142857 |
| Putamen_L | 7992 | 16 | 0,2002002 |
| Insula_L | 14864 | 24 | 0,16146394 |

Supplementary table 2 Areas affected by stroke lesions associated with Acute Myocardial injury

| ROI NAME | ROI VOLUME | LESION OVERLAP VOLUME | LESION % ROI VOLUME |
| --- | --- | --- | --- |
| Putamen_R | 8496 | 288 | 3,389830508 |
| External capsule L | 3600 | 112 | 3,111111111 |
| Retrolenticular part of internal capsule L | 2488 | 64 | 2,572347267 |
| External capsule R | 3728 | 56 | 1,502145923 |
| Posterior limb of internal capsule L | 3816 | 56 | 1,467505241 |
| Inferior fronto-occipital fasciculus R | 2104 | 8 | 0,380228137 |
| Pallidum_R | 2240 | 8 | 0,357142857 |
| Putamen_L | 7992 | 16 | 0,2002002 |
| Insula_L | 14864 | 24 | 0,16146394 |

Supplementary table 2 Areas affected by stroke lesions associated with Acute Myocardial injury

| ROI NAME | ROI VOLUME | DYSCONNECTION VOLUME | % |
| --- | --- | --- | --- |
| Thal_LGN_L | 344 | 64 | 18,60465116 |
| Posterior thalamic radiation (include optic radiation) L | 3824 | 680 | 17,78242678 |
| Posterior corona radiata R | 3616 | 440 | 12,16814159 |
| Posterior corona radiata L | 3568 | 328 | 9,192825112 |
| Superior longitudinal fasciculus R | 6600 | 408 | 6,181818182 |
| Inferior fronto-occipital fasciculus R | 2104 | 104 | 4,942965779 |
| Body of corpus callosum | 13816 | 392 | 2,837290098 |
| External capsule R | 3728 | 104 | 2,789699571 |
| Fornix (cres) / Stria terminalis L | 1176 | 32 | 2,721088435 |
| Inferior fronto-occipital fasciculus L | 1936 | 40 | 2,066115702 |
| Putamen_R | 8496 | 144 | 1,694915254 |
| Retrolenticular part of internal capsule L | 2488 | 40 | 1,607717042 |
| Occipital_Sup_R | 11304 | 176 | 1,556970984 |
| Splenium of corpus callosum | 12344 | 152 | 1,231367466 |
| Posterior thalamic radiation (include optic radiation) R | 3896 | 40 | 1,026694045 |
| Fornix (cres) / Stria terminalis R | 1096 | 8 | 0,729927007 |
| Superior corona radiata R | 7360 | 48 | 0,652173913 |
| Precuneus_R | 26120 | 104 | 0,398162328 |
| Cuneus_R | 11392 | 40 | 0,351123596 |
| Cingulate_Post_L | 3704 | 8 | 0,215982721 |
| Hippocampus_L | 7456 | 16 | 0,214592275 |
| Superior corona radiata L | 7392 | 8 | 0,108225108 |
| Hippocampus_R | 7568 | 8 | 0,105708245 |
| Paracentral_Lobule_L | 10792 | 8 | 0,074128984 |
| Temporal_Inf_R | 28456 | 16 | 0,056227158 |
| Precuneus_L | 28224 | 8 | 0,028344671 |
| Temporal_Mid_R | 35272 | 8 | 0,02268088 |

Supplementary Table 3 Areas of disconnections associated with the occurrence of acute myocardial injury.
