# Supplementary figures and images for: Neuroimaging of acute myocardial injury in stroke: insights into brain lesion locations and network disconnections
Source: Front Neurol. 2026 Feb 11;17:1719600. doi: 10.3389/fneur.2026.1719600 (PMC12932174; doi:10.3389/fneur.2026.1719600)

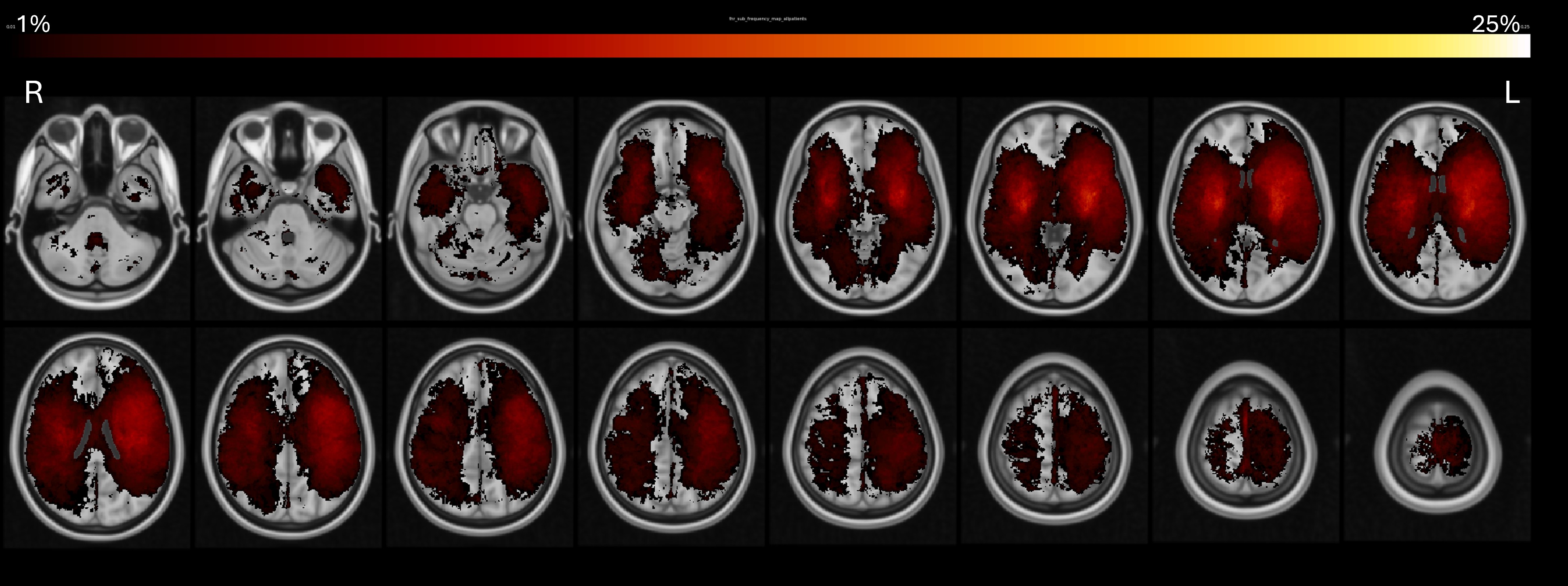

Supplement: Supplementary file 4 [file Image_1.jpeg]

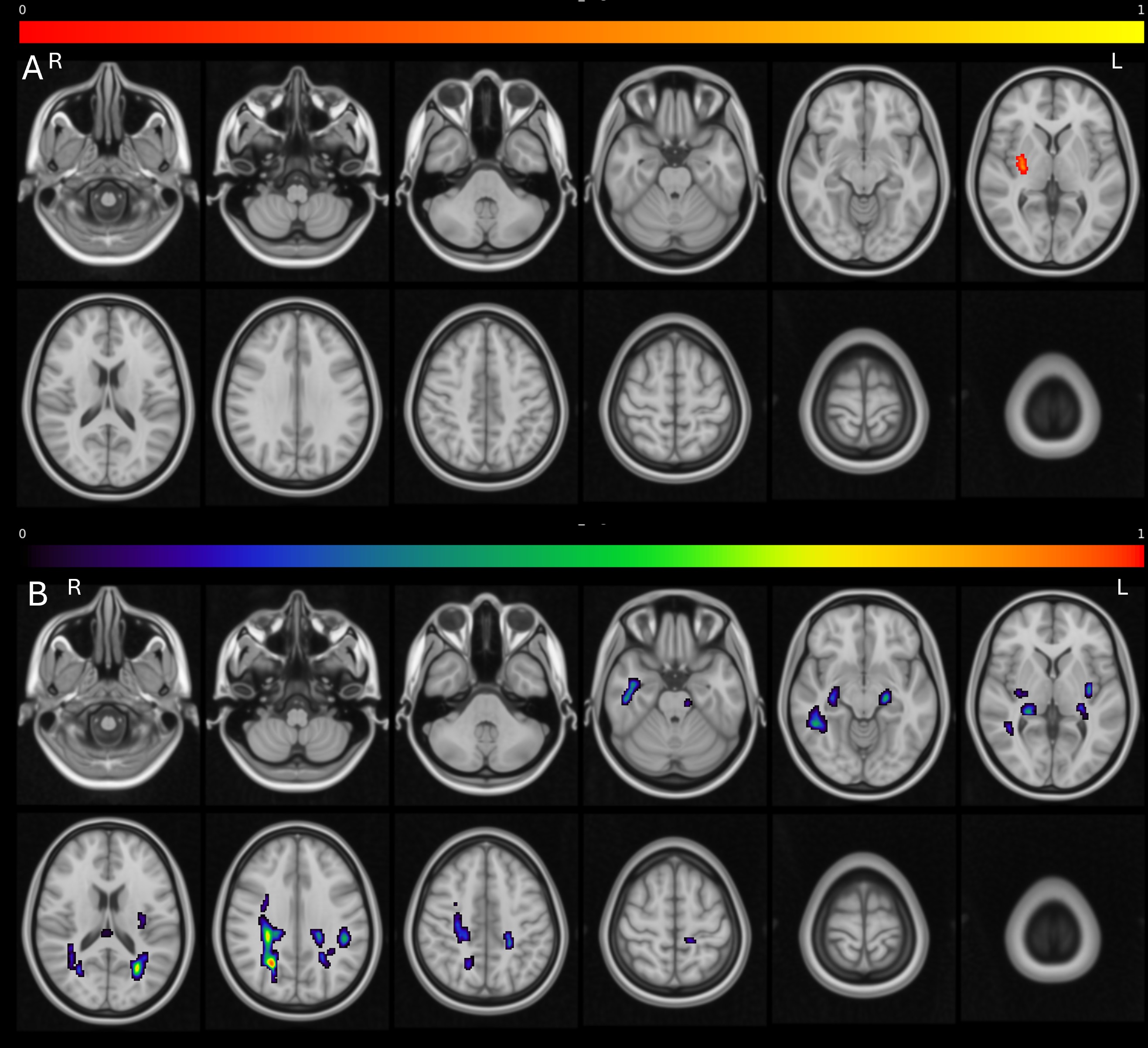

Supplement: Supplementary file 5 [file Image_2.jpeg]

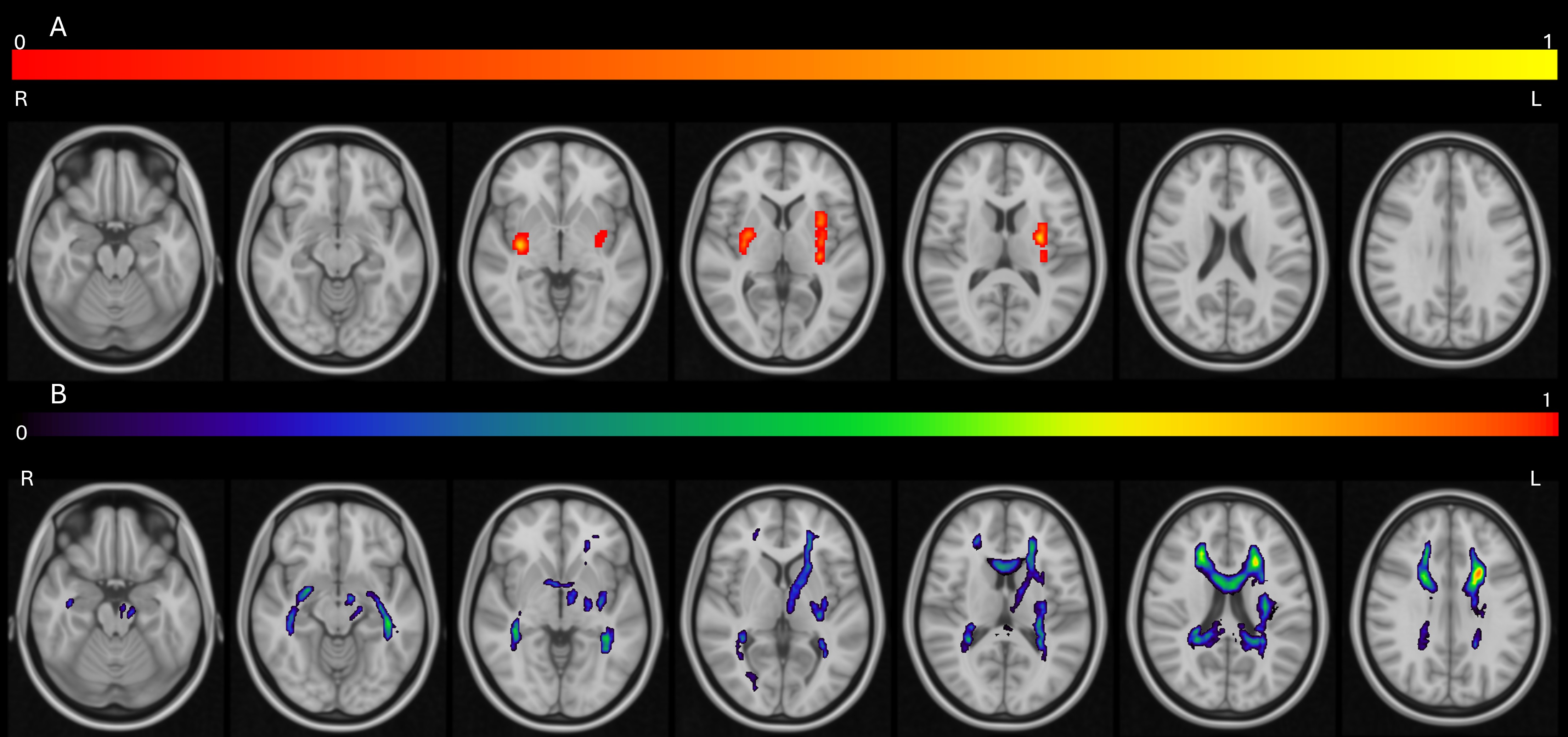

Supplement: Supplementary file 6 [file Image_3.jpeg]
